# Association Analysis of Grain-setting Rates in Apical and Basal Spikelets in Bread Wheat (*Triticum aestivum L*.)

**DOI:** 10.3389/fpls.2015.01029

**Published:** 2015-11-20

**Authors:** Jie Guo, Yong Zhang, Weiping Shi, Boqiao Zhang, Jingjuan Zhang, Yanhao Xu, Xiaoming Cheng, Kai Cheng, Xueyong Zhang, Chenyang Hao, Shunhe Cheng

**Affiliations:** ^1^Key Laboratory of Wheat Biology and Genetic Improvement for Low and Middle Yangtze Valley (Ministry of Agriculture), Lixiahe Agricultural Institute of Jiangsu ProvinceYangzhou, China; ^2^Key Laboratory of Crop Gene Resources and Germplasm Enhancement, Ministry of Agriculture/The National Key Facility for Crop Gene Resources and Genetic Improvement/Institute of Crop Science, Chinese Academy of Agricultural SciencesBeijing, China; ^3^Institute for Chemical Ecology, Shanxi Agricultural UniversityTaigu, China; ^4^Agricultural Science, School of Veterinary and Life Sciences, Murdoch UniversityMurdoch, WA, Australia; ^5^Hubei Collaborative Innovation Centre for Grain Industry/College of Agriculture, Yangtze UniversityJingzhou, China

**Keywords:** bread wheat, released cultivars, grain numbers in apical spikelets, grain numbers in basal spikelets, association analysis

## Abstract

The rates of grain-setting in apical and basal spikelets in wheat directly affect the kernel number per spike (KNPS). In this study, 220 wheat lines from 18 Chinese provinces and five foreign countries were used as a natural population. Phenotypic analysis showed differences in grain-setting rates between apical and basal spikelets. The broad-sense heritability of grain-setting rate in apical spikelets (18.7–21.0%) was higher than that for basal spikelets (9.4–16.4%). Significant correlations were found between KNPS and grain numbers in apical (*R*^2^ = 0.40–0.45, *P* < 0.01) and basal (*R*^2^ = 0.41–0.56, *P* < 0.01) spikelets. Seventy two of 106 SSR markers were associated with grain setting, 32 for apical spikelets, and 34 for basal spikelets. The SSR loci were located on 17 chromosomes, except 3A, 3D, 4A, and 7D, and explained 3.7–22.9% of the phenotypic variance. Four markers, *Xcfa2153-1A*_**202**_, *Xgwm186-5A*_**118**_, *Xgwm156-3B*_**319**_, and *Xgwm537-7B*_**210**_, showed the largest effects on grain numbers in apical and basal spikelets. High grain numbers in apical and basal spikelets were associated with elite alleles. Ningmai 9, Ning 0569, and Yangmai 18 with high grain-setting rates carried larger numbers of favorable alleles. Comparison of grain numbers in basal and apical spikelets of 35 Yangmai and Ningmai lines indicated that the Ningmai lines had better grain-setting rates (mean 21.4) than the Yangmai lines (16.5).

## Introduction

Wheat is one of the most important cereal crops in the world. As the third-largest crop in China, the average grain yield of wheat has reached 4986 kg/ha, and the maximum was more than 12,000 kg/ha (National Bureau of Statistics of China, http://data.stats.gov.cn). Improved grain yield is still the most important objective in wheat breeding. Spike number per square meter, kernel number per spike (KNPS) and thousand grain weight (TGW) are important grain yield components. At a fixed spike number per square meter and fixed TGW, improvements in grain yield will depend largely on increasing KNPS (Slafer and Andrade, 1989; Fischer, 1993, 2008, 2011; Magrin et al., 1993). The numbers of grain produced in apical and basal spikelets become key factors for achieving high kernel numbers per spike (Arisnabarreta and Miralles, 2006; Acreche et al., 2008).

The physiological processes of grain formation in apical and basal splikelets include floret differentiation, grain development, and floret degeneration. The number of fertile florets is the key factor determining KNPS (Stockman et al., 1983; Sibony and Pinthus, 1988; González et al., 2003; Shitsukawa et al., 2009). Flower development in wheat spikes is not synchronized. The central spikelets develop first, followed by the distal spikelets. Florets that developed first can continue to develop and form seeds whereas the later florets are more subject to non-viability and loss of fertility (Kirby, 1988; Bancal, 2009; Ferrante et al., 2013). Moreover, floret fertility varies with position on the spike (González-Navarro et al., 2015). Previous studies elucidated causes of low fertility in apical and basal spikelets from the perspective of physiology. Floret differentiation and development are also influenced by environmental factors, and grain-setting rates are affected by various factors such as cultivation practices (Fischer, 1993; Demotes-Mainard et al., 1999; Ferrante et al., 2010), temperature (Dreccer et al., 2014), and light (González et al., 2003).

Low floret fertility in the apical and basal regions of the ear is a very common biological phenomenon in cereal crops, as shown by the “bald” phenomenon in maize (Satoh-Nagasawa et al., 2006; Meng et al., 2007; Gallavotti et al., 2011). More detailed studies have been conducted in rice (Yamagishi et al., 2004; Li et al., 2009; Cheng et al., 2011; Tan et al., 2011; Akter et al., 2014). Gene *SP1* was shown to be a PTR transporter following map-based cloning and mutation caused severe degradation of basal spikelets (Li et al., 2009). A recessive gene for apical floret degradation in rice was fine mapped (Akter et al., 2014). There are almost no reports on isolation of genes involved in apical spikelet degradation. In wheat, there are few reports on grain-setting rates of apical and basal spikelets. However, there are many studies on spikelet infertility (Slafer and Andrade, 1989; Li et al., 2007; Ma et al., 2007; Wu et al., 2012; Xu et al., 2014). Those reports showed that additive effects play a major role in floret fertility through the study of heritability of yield-related traits and the mode of gene function using two crosses of common wheat (Salman and Heyne, 1987). QTLs associated with floret sterility were detected in 13 chromosomal regions using a recombinant inbreb line (RIL) population derived from the cross Nanda 2419 × Wangshuibai and an immortalized F_2_(IF_2_) population (Ma et al., 2007). QTLs *QSspn.nau-4A* and *QSspn.nau-5A* were detected in both populations. One QTL for floret infertility was detected in the interval *Xwmc112*-*Xbarc168* in chromosome 2D using a Xiaoyan 54 × Jing 411 RIL population grown under different levels of nitrogen and phosphorus (Xu et al., 2014). Reduced height (*Rht*) genes also indirectly affect grain-setting rates (Miralles et al., 1998; Álvaro et al., 2008). One study showed that semi-dwarf varieties with *Rht* genes had significantly increased kernel numbers per spike, especially kernel numbers in the apical and basal spikelets (Miralles et al., 1998). Therefore, genetic information on control of grain-setting rates is of significant theoretical and applied value in understanding the underlying mechanisms.

In this study, we used 220 wheat varieties from 18 Chinese provinces and five foreign countries as a natural population to analyse the heritability of grain-setting rates in apical and basal spikelets in different environments. SSR loci associated with grain-setting rates in apical and basal spikelets were studied using association analysis. To some extent the study revealed possible reasons for significant differences in grain-setting rate between two popular series of wheat varieties, namely Yangmai and Ningmai cultivars, which have been widely cultivated in the lower Yangtze River region. The study provides evidence that increased KNPS can be achieved by improvement of grain-setting rates in the apical and basal spikelets. Our association results indicate that marker-assisted selection can be used to increase levels of grain setting in wheat.

## Materials and methods

### Plant materials

A natural population formed by 220 wheat varieties was used for association analysis. There were 204 varieties from 18 provinces in China and seven foreign varieties. The origins of nine varieties were unknown. The seven foreign varieties from Italy (3), the U.S.A. (1), Mexico (1), Chile (1), and Japan (1) are historic donors that made important contributions to wheat breeding in China (Zhuang, 2003). The 18 provincial origins for the Chinese varieties were Jiangsu (65), Henan (25), Shandong (19), Shaanxi (18), Sichuan (16), Anhui (13) Hunan (10), Hubei (6), Beijing (7), Hebei (7), Gansu (4), Zhejiang (3), Fujian (3), Shanxi (3), Heilongjiang (2), Jiangxi (1), Guizhou (1), and Yunnan (1). All grow normally in the Yangtze River region. Variety details are provided in Table S1.

### Phenotyping and genotyping

The 220 wheat varieties were planted in Jingzhou (JZ), Hubei province, and Yangzhou (YZ), Jiangsu province in 2013 and 2014. The notations 13JZ, 13YZ, 14JZ, and 14YZ represent the four experiments conducted over 2 years. Field trial entries were randomized and three replicates were used for each variety per experiment. Each plot was 1.33 m long and 0.75 m wide. Seeds were evenly sown in three rows at approximately 40 seeds per row. To achieve a plant density of 30 plants per row some seedlings were removed. At the maturity stage, plants in the middle row of each plot were selected for the measurement of KNPS. Kernel number for each spikelet from the apex downwards was recorded as GNAS1, GNAS2, and GNAS3, and kernel number for each spikelet from the base upwards was recorded as GNBS1, GNBS2, and GNBS3 (Figure S1).

Genomic DNA was extracted using the CTAB method (Sharp et al., 1989) and 106 SSR markers distributed on all 21 chromosomes were used. Among the 106 SSR markers, 21 markers were previously reported to be associated with the KNPS (Zhang et al., 2012) and 17 SSR markers were chosen based on linkage with KNPS and spikelet infertility (Guo et al., 2015).

### Data analysis

DNA fragment sizes were detected using an ABI 3730 DNA sequencer (Applied Biosystems, Foster City, USA) and software Genemapper V 3.7 (Applied Biosystems; http://www.appliedbiosystems.com.cn/) was used for data recording. SPSS 21.0 (Genetic Diversity Evaluation http://www.brothersoft.com/ibm-spss-statistics-469577.html) was used in statistical analysis of phenotypic variation. PowerMarker V 3.25 software was used for evaluation of genetic diversity (Liu and Muse, 2005) and Structure V 2.3.2 was used for evaluation of genetic structure of the natural population using the 106 SSR markers (Pritchard et al., 2000). The number of subsets was determined with the ΔK model (Evanno et al., 2005). SPAGeDi software was used for calculation of genetic covariance between pairs of individuals (Loiselle et al., 1995; Yu et al., 2005). Based on the model of Q + K (Zhang et al., 2010b), association analysis of grain-setting rate and SSR markers was performed using TASSEL 2.1 (Bradbury et al., 2007) (http://www.maizegenetics.net/). The genetic effects of favorable alleles were calculated using ANOVA program. Based on the data of phenotype and kinship between individuals, broad heritabilities in different environments were calculated according to the formula of *h*^2^ $=\sigma_{\text{a}}^{2}$/($\sigma_{\text{a}}^{2}+\sigma_{\text{e}}^{2}$) using TASSEL 2.1, where $\sigma_{\text{a}}^{2}$ was genetic variance and $\sigma_{\text{e}}^{2}$ represented the residual variance.

## Results

### Phenotypic assessments

Related phenotypes for grain-setting rates in apical and basal spikelets (GNAS1, GNAS2, GNAS3, GNBS1, GNBS2, GNBS3, and KNPS) were investigated in four environments (13JZ, 13YZ, 14JZ, and 14YZ). The average coefficient of variation for each trait within the population was between 10.89 and 87.85%, indicating wide variation in grain-setting rate in the natural population, especially in regard to GNBS1. The average grain numbers per spike for GNAS1, GNAS2, and GNAS3 in the four environments were 1.39, 1.54, and 1.78, whereas those for GNBS1, GNBS2, GNBS3, were 0.46, 1.44, and 2.60, respectively (Table 1). The results indicated differences in grain-setting rates between apical and basal spikelets. Similar numbers of grains were produced in all apical spikelets. However, a significant factor was that the grain-setting rate was obviously low for GNBS1.

**Table 1:** **Descriptive statistics for seven phenotypic traits assessed in this study**.

**Trait**	**13JZ**				**13YZ**				**14JZ**				**14YZ**				**Average**				***h*^2^^*c*^**
	**Mean ± SD^a^**	**Min**	**Max**	**CV (%)^b^**	**Mean ± SD**	**Min**	**Max**	**CV (%)**	**Mean ± SD**	**Min**	**Max**	**CV (%)**	**Mean ± SD**	**Min**	**Max**	**CV (%)**	**Mean ± SD**	**Min**	**Max**	**CV (%)**	
GNAS1	1.33 ± 0.33	0.40	3.13	25.09	1.40 ± 0.35	0.35	2.55	25.24	1.37 ± 0.28	0.75	2.15	20.18	1.49 ± 0.33	0.13	2.27	22.04	1.39 ± 0.28	0.47	2.34	20.10	18.68
GNAS2	1.42 ± 0.32	0.47	2.21	22.62	1.48 ± 0.36	0.65	2.45	24.38	1.63 ± 0.25	0.95	2.20	15.04	1.67 ± 0.31	0.30	2.63	18.90	1.54 ± 0.26	0.69	2.29	16.93	20.49
GNAS3	1.69 ± 0.34	0.60	2.69	19.89	1.73 ± 0.36	0.55	3.00	20.89	1.85 ± 0.19	1.10	2.45	10.51	1.87 ± 0.32	0.37	2.87	17.10	1.78 ± 0.26	0.98	2.60	14.68	21.00
GNBS1	0.25 ± 0.41	0.00	2.40	162.67	0.33 ± 0.47	0.00	2.90	142.98	0.38 ± 0.42	0.00	2.10	110.71	0.94 ± 0.71	0.00	3.00	75.37	0.46 ± 0.40	0.00	1.96	87.85	9.44
GNBS2	1.10 ± 0.79	0.00	3.90	71.75	1.25 ± 0.81	0.00	3.75	64.83	1.37 ± 0.69	0.00	3.05	50.60	2.12 ± 0.78	0.03	3.73	36.85	1.44 ± 0.65	0.05	3.30	45.24	12.40
GNBS3	2.37 ± 0.74	0.00	4.10	31.19	2.55 ± 0.70	0.20	4.15	27.28	2.55 ± 0.59	0.45	3.65	23.18	2.99 ± 0.58	0.20	4.23	19.45	2.60 ± 0.57	0.43	3.90	21.89	16.37
KNPS	54.44 ± 7.31	33.17	94.23	13.43	57.81 ± 7.56	37.60	95.40	13.07	51.45 ± 5.61	36.95	68.75	10.90	58.08 ± 6.77	42.13	95.50	11.65	55.41 ± 6.03	38.53	88.32	10.89	18.36

a *SD, standard deviation*.

b *CV, coefficient of variation*.

c *h^2^, broad-sense heritability*.

Broad-sense heritabilities for apical spikelets, namely GNAS1, GNAS2, GNAS3 were 18.68, 20.49, and 21.00%, respectively, whereas for basal spikelets the corresponding values were 9.44, 12.40, and 16.37% for GNBS1, GNBS2, and GNBS3, respectively (Table 1). Broad-sense heritabilities for GNAS1 and GNBS1 were lower than for other spikelets, indicating that the terminal spikelets were subject to greater environmental influence. In general, the heritabilities for apical spikelets were higher than for basal spikelets and increased from the apex downwards and from the base upwards.

### Correlation between grain-setting rates in apical and basal spikelets, and kernel number per spike

Analyses of regression between KNPS and total kernel number of the three spikelets from apical or basal spikes were performed in all four environments. Strong positive correlation (*R*^2^) of 0.40–0.45 (*P* < 0.01) and 0.41–0.56 (*P* < 0.01) in apical and basal spikelets, respectively, occurred in all four environments.

**Figure 1:**
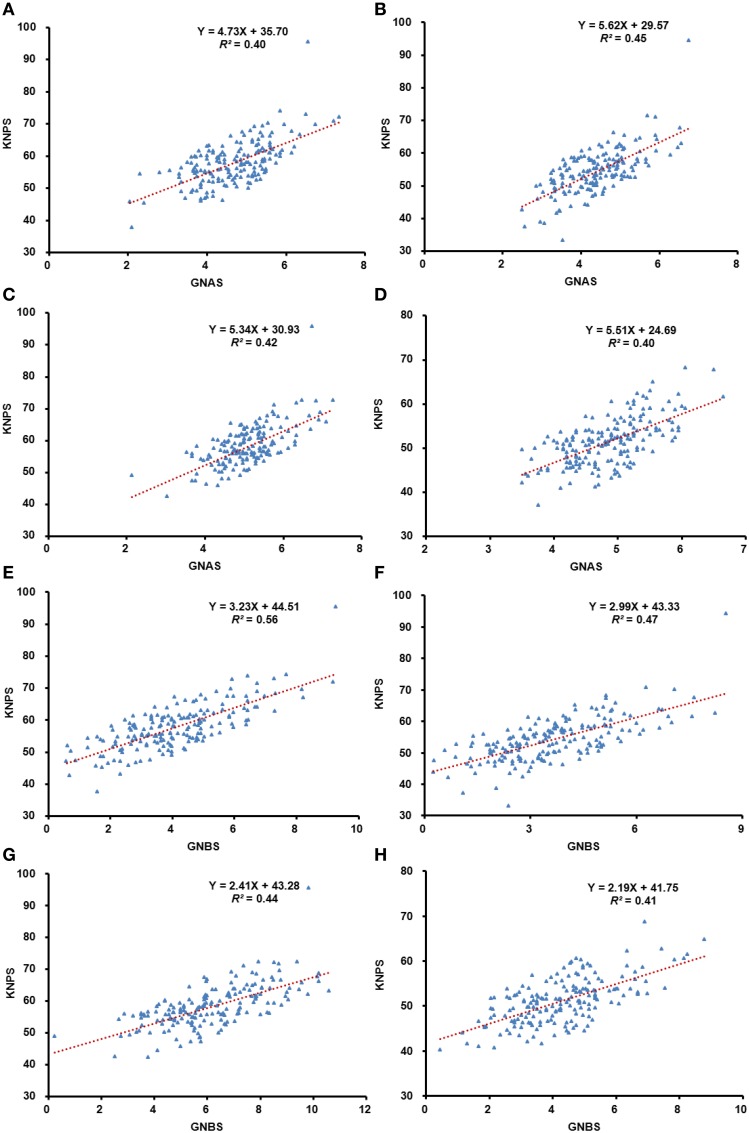
**Analysis of regression between grain-setting in the whole spike, and apical and basal spikelets, respectively**. Blue dots represent the phenotypic value and the red dotted lines represent the predicted regression line. The correlation between grain-setting in apical spikelets and kernel number per spike in 13JZ **(A)**, 13YZ **(B)**, 14JZ **(C)**, and 14YZ **(D)**; Correlations between the grain-setting in basal spikelets and kernel number per spike in 13JZ **(E)**, 13YZ **(F)**, 14JZ **(G)**, and 14YZ **(H)**.

Correlation analysis of kernel number was also performed between the apical and basal spikelets, as well as with KNPS. There were significant correlations (*P* < 0.01) in all comparisons (Figure 1, Table S2). On average, correlations between KNPS and grain number of apical and basal spikelets were in the range of 0.31–0.48. The average correlation of grain number within apical spikelets was in the range of 0.87–0.94 compared to 0.69–0.89 within basal spikelets.

### Allelic diversity and genetic structure analysis

Eight hundred and seventy nine allelic variations were detected in the population using 106 SSR markers and the range in major allele frequency (MAF) was from 0.164 to 0.986, with an average of 0.541. The range in allele number per locus was 2–24, with an average of 8.3. The range of polymorphism information content (PIC) was from 0.027 to 0.908, with an average of 0.555 (Table S3) indicating that the natural population has a high level of genetic diversity.

**Figure 2:**
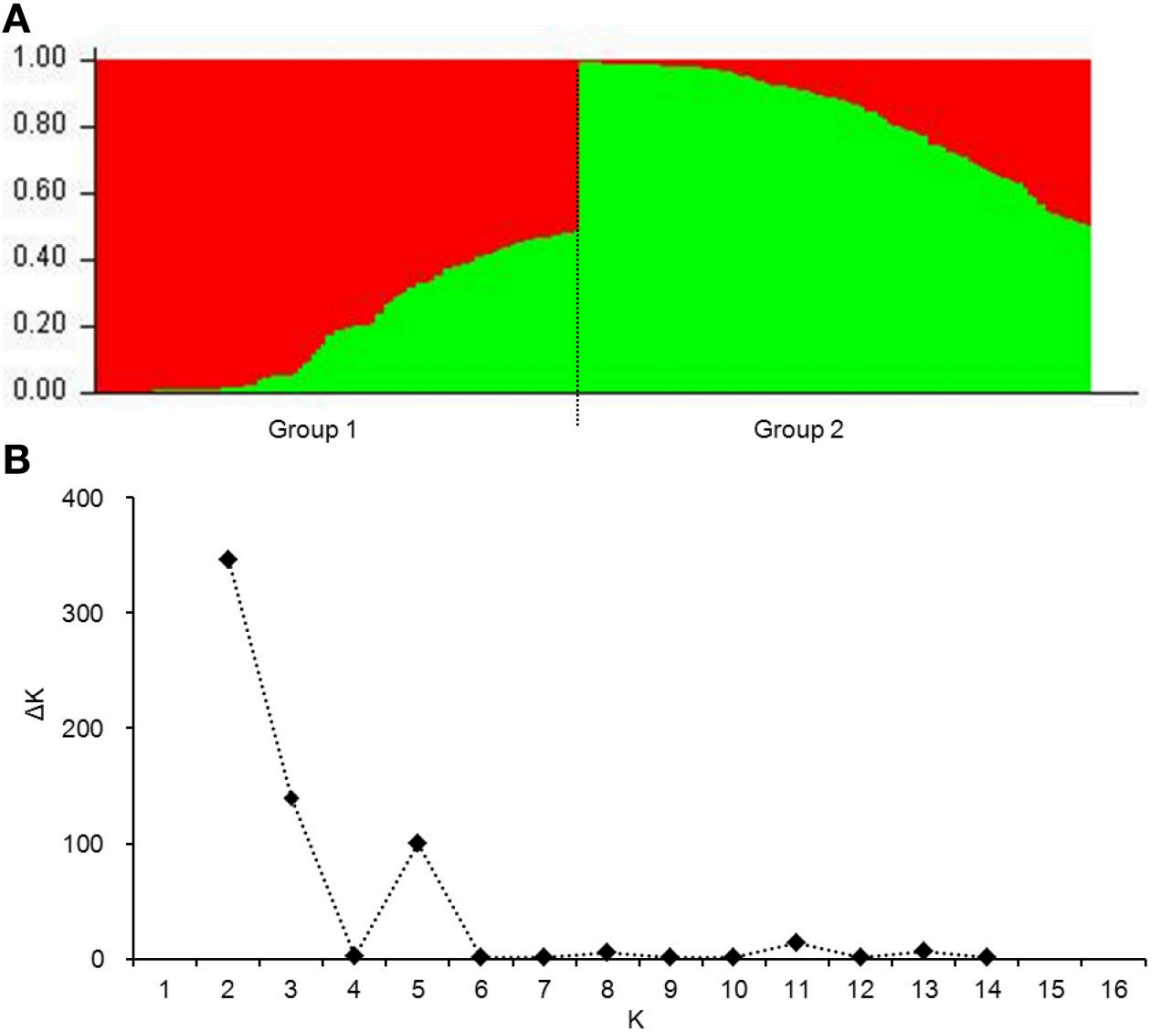
**Population structure of 220 wheat cultivars based on 106 genome-wide SSR markers**. **(A)**, genetic structure produced by Structure V2.3.2; **(B)**, number of sub-populations estimated by ΔK at a range of *K*-values.

To eliminate spurious associations, genetic structure (*Q*-value) and the individual kinship coefficient (*K*-value) were evaluated. Two subgroups were evident (Figure 2A). The maximum value of ΔK at the point of *K* = 2 further demonstrated two subgroups (Figure 2B). For the whole population, 76.5% of individual kinship coefficients were in the range of 0–0.05 (Figure S3). Low coefficients between individuals indicate no or little relationship between them.

### Association analysis between grain setting-related phenotypes and SSR markers

In the association analysis for grain-setting rates of apical and basal spikelets and KNPS using 106 SSR markers (including 879 alleles), 72 significant association signals were detected at 36 SSR loci, including 32 signals for apical grain-setting rates at 18 SSR loci, 34 signals for the basal grain-setting rate at 20 SSR loci, and six signals for KNPS at five SSR markers. The range in phenotypic explanation rates was 3.71–22.93%. The 36 SSR loci were distributed on chromosomes except 3A, 3D, 4A, and 7D (Table 2, Figure S2). Significant association signals detected in more than two environments included *Xcfa2153-1A* (GNAS2 and GNAS3), *Xgwm148-2B* (GNAS1), *Xgwm102-2D* (GNAS2), *Xgwm108-3B* (GNBS3), *Xbarc56-5A* (GNBS3), *Xgwm126-5A* (GNBS1), *Xgwm186-5A* (GNBS1), *Xgwm415-5A* (GNBS3), *Xgwm55-6D* (GNBS3), *Xwmc168-7A* (GNBS1), and *Xgwm537-7B* (KNPS, GNBS2, and GNBS3). Thirteen SSR markers were significantly associated with more than two phenotypes including *Xcfa2153-1A* (GNAS1, GNAS2, and GNAS3), *Xgwm403-1B* (GNAS3 and GNBS3), *Xwmc147-1D* (GNAS1, GNBS1, and GNBS2), *Xgwm312-2A* (GNAS2 and GNAS3), *Xgwm148-2B* (GNAS1, GNAS2, and GNAS3), *Xgwm429-2B* (GNAS1 and GNAS3), *Xgwm102-2D* (GNAS1, GNAS2, and GNAS3), *Xgwm156-3B* (GNAS2 and GNBS2), *Xgwm149-4B* (GNAS3 and GNBS1), *Xcfd52-5D* (GNAS1, GNAS2, and GNAS3), *Xgwm190-5D* (GNAS3 and GNBS3), *Xgwm292-5D* (KNPS and GNAS3), and *Xgwm537-7B* (KNPS, GNBS1, GNBS2, and GNBS3). *Xgwm102-2D, Xgwm108-3B, Xgwm186-5A, Xgwm415-5A, Xcfd52-5D*, and *Xgwm55-6D* were associated with grain-setting rate QTLs in previous studies (Peng et al., 2003; Zhang et al., 2010a, 2012; Guo et al., 2015; Table 2).

**Table 2:** **Seventy-two significant association signals (*P* < 0.01) involving 36 SSR loci and seven phenotypic traits**.

**Trait**	**Locus**	**Allele size (bp)**	**Chr**.	**Environment**	***P*-value**	***R*^2^**	**QTL reported**	**Trait**	**Locus**	**Allele size (bp)**	**Chr**.	**Environment**	***P*-value**	***R*^2^**	**QTL reported**
KNPS	*Xgwm268*	200–246	1B	14YZ	6.2 × 10^−3^	22.93		GNAS3	*Xgwm190*	204–216	5D	14YZ	3.7 × 10^−3^	8.82	
	*Xgwm292*	196–216	5D	14YZ	7.7 × 10^−3^	11.54			*Xgwm292*	196–216	5D	13JZ	7.2 × 10^−3^	8.60	
	*Xgwm219*	148–188	6B	13JZ	8.9 × 10^−3^	9.82		GNBS1	*Xwmc147*	140–154	1D	14JZ	4.9 × 10^−3^	8.84	
	*Xgwm46*	142–186	7B	14YZ	9.2 × 10^−3^	17.04			*Xgwm285*	206–304	3B	13YZ	8.2 × 10^−4^	20.49	
	*Xgwm537*	204–228	7B	13YZ	5.5 × 10^−3^	11.35			*Xgwm149*	149–171	4B	13JZ	1.8 × 10^−3^	11.77	
				14YZ	4.8 × 10^−3^	10.79			*Xgwm194*	127–135	4D	13JZ	1.4 × 10^−5^	13.48	Zhang et al., 2010a
GNAS1	*Xcfa2153*	168–224	1A	13YZ	2.1 × 10^−3^	18.69			*Xgwm126*	191–199	5A	13JZ	1.1 × 10^−4^	13.31	
	*Xgwm413*	84–118	1B	13JZ	1.6 × 10^−3^	16.87						14JZ	3.8 × 10^−4^	8.30	
	*Xwmc147*	140–154	1D	13JZ	1.5 × 10^−3^	10.11			*Xgwm186*	98–150	5A	13JZ	5.2 × 10^−3^	11.95	Peng et al., 2003
	*Xgwm148*	136–166	2B	14JZ	6.2 × 10^−3^	11.35						13YZ	8.3 × 10^−5^	19.44	Guo et al., 2015
				14YZ	2.6 × 10^−3^	14.77			*Xgwm234*	225–249	5B	13JZ	1.4 × 10^−3^	16.43	Guo et al., 2015
	*Xgwm429*	197–217	2B	13JZ	3.1 × 10^−3^	10.49			*Xgwm212*	78–100	5D	13YZ	6.1 × 10^−3^	8.31	
	*Xgwm102*	134–154	2D	13YZ	6.1 × 10^−3^	14.55	Guo et al., 2015		*Xwmc168*	303–327	7A	13JZ	5.1 × 10^−3^	10.21	
	*Xgwm484*	131–185	2D	13YZ	5.1 × 10^−3^	21.95						13YZ	7.5 × 10^−4^	15.53	
	*Xcfd52*	278–284	5D	14YZ	2.3 × 10^−3^	9.11	Zhang et al., 2012		*Xgwm537*	204–228	7B	14JZ	5.3 × 10^−3^	11.39	
	*Xgwm169*	181–203	6A	13YZ	9.1 × 10^−3^	9.92		GNBS2	*Xwmc147*	140–154	1D	14JZ	8.7 × 10^−3^	8.59	
	*Xgdm127*	184–188	6D	13JZ	3.7 × 10^−3^	4.06	Zhang et al., 2012		*Xgwm156*	299–321	3B	13JZ	9.0 × 10^−3^	13.98	
GNAS2	*Xcfa2153*	168–224	1A	13YZ	4.4 × 10^−3^	16.58			*Xgwm537*	204–228	7B	13YZ	1.6 × 10^−3^	15.17	
				14YZ	9.8 × 10^−3^	15.41						14JZ	6.4 × 10^−3^	11.95	
	*Xgwm312*	186–252	2A	14YZ	1.1 × 10^−3^	20.24		GNBS3	*Xgwm403*	130–144	1B	14YZ	4.1 × 10^−5^	16.87	
	*Xgwm148*	136–166	2B	14YZ	1.3 × 10^−3^	14.71			*Xgwm337*	168–198	1D	14YZ	7.3 × 10^−3^	14.82	Zhang et al., 2012
	*Xgwm102*	134–154	2D	13JZ	5.1 × 10^−3^	15.68	Guo et al., 2015		*Xgwm501*	160–180	2B	14YZ	4.7 × 10^−3^	13.20	
				13YZ	4.2 × 10^−3^	15.03			*Xgwm108*	105–131	3B	13JZ	4.1 × 10^−3^	8.05	Zhang et al., 2012
	*Xgwm156*	299–321	3B	13JZ	8.0 × 10^−3^	11.80						13YZ	2.4 × 10^−3^	9.27	
	*Xcfd52*	278–284	5D	14YZ	4.0 × 10^−4^	10.20	Zhang et al., 2012					14JZ	3.4 × 10^−3^	9.57	
GNAS3	*Xcfa2153*	168–224	1A	13YZ	7.9 × 10^−4^	20.00			*Xbarc56*	117–137	5A	13YZ	9.5 × 10^−3^	5.98	
				14YZ	1.4 × 10^−3^	19.44						14JZ	1.1 × 10^−3^	10.44	
	*Xwmc24*	125–145	1A	14YZ	7.8 × 10^−3^	10.31	Zhang et al., 2012		*Xgwm415*	130–132	5A	13JZ	4.2 × 10^−3^	9.36	Guo et al., 2015
	*Xgwm403*	130–144	1B	14YZ	6.7 × 10^−3^	8.94						13YZ	6.8 × 10^−3^	3.71	
	*Xgwm312*	186–252	2A	14YZ	7.2 × 10^−4^	21.23						14JZ	5.5 × 10^−3^	7.27	
	*Xgwm95*	105–129	2A	13YZ	6.9 × 10^−3^	12.13			*Xgwm499*	96–146	5B	14YZ	2.6 × 10^−3^	14.16	
	*Xgwm148*	136–166	2B	14YZ	3.5 × 10^−4^	16.65			*Xgwm190*	204–216	5D	14YZ	6.0 × 10^−4^	13.51	
	*Xgwm429*	197–217	2B	13YZ	1.3 × 10^−3^	8.83			*Xgwm55*	118–138	6D	13YZ	5.5 × 10^−3^	13.31	Guo et al., 2015
	*Xgwm102*	134–154	2D	13YZ	4.0 × 10^−3^	16.48	Guo et al., 2015					14JZ	8.6 × 10^−3^	14.23	
	*Xgwm149*	149–171	4B	13YZ	9.8 × 10^−3^	8.88			*Xgwm537*	204–228	7B	13JZ	3.5 × 10^−3^	12.21	
	*Xcfd52*	278–284	5D	14YZ	1.3 × 10^−4^	12.11	Zhang et al., 2012					13YZ	1.2 × 10^−3^	14.64	

### Favorable alleles and their genetic effects

Favorable alleles and their genetic effects for each locus were further analyzed following association analysis (Table 2, Table S4). The largest genetic effect for KNPS was associated with *Xgwm537-7B*_**210**_ (2.18, 14YZ). *Xcfa2153-1A*_**202**_ had the largest genetic effect on apical spikelets, the values of GNAS1, GNAS2, GNAS3 were 0.31 (13YZ), 0.20/0.29 (13YZ and 14YZ), and 0.20/0.34 (13YZ and 14YZ), respectively, while basal spikelets genetic effects for GNBS1, GNBS2, and GNBS3 were 0.23 (13YZ) at *Xgwm186-5A*_**118**_, 0.87 (13JZ) at *Xgwm156-3B*_**319**_, and 0.31/0.33 (13JZ and 13YZ) at *Xgwm537-7B*_**210**_, respectively. Favorable alleles with frequencies greater than 50% were *Xgwm403-1B*_**136**_ (GNAS3 and GNBS3), *Xgwm102-2D*_**144**_ (GNAS1, GNAS2, and GNAS3), *Xbarc56-5A*_**131**_ (GNBS3), *Xgwm499-5B*_**124**_ (GNBS3), *Xcfd52-5D*_**282**_ (GNAS1, GNAS2, and GNAS3), *Xgwm190-5D*_**210**_ (GNBS3), *Xgwm212-5D*_**96**_ (GNBS1), and *Xgwm292-5D*_**208**_ (KNPS and GNAS3). The high frequencies suggest that varieties possessing these alleles had been subjected to strong selection pressure in modern breeding.

**Figure 3:**
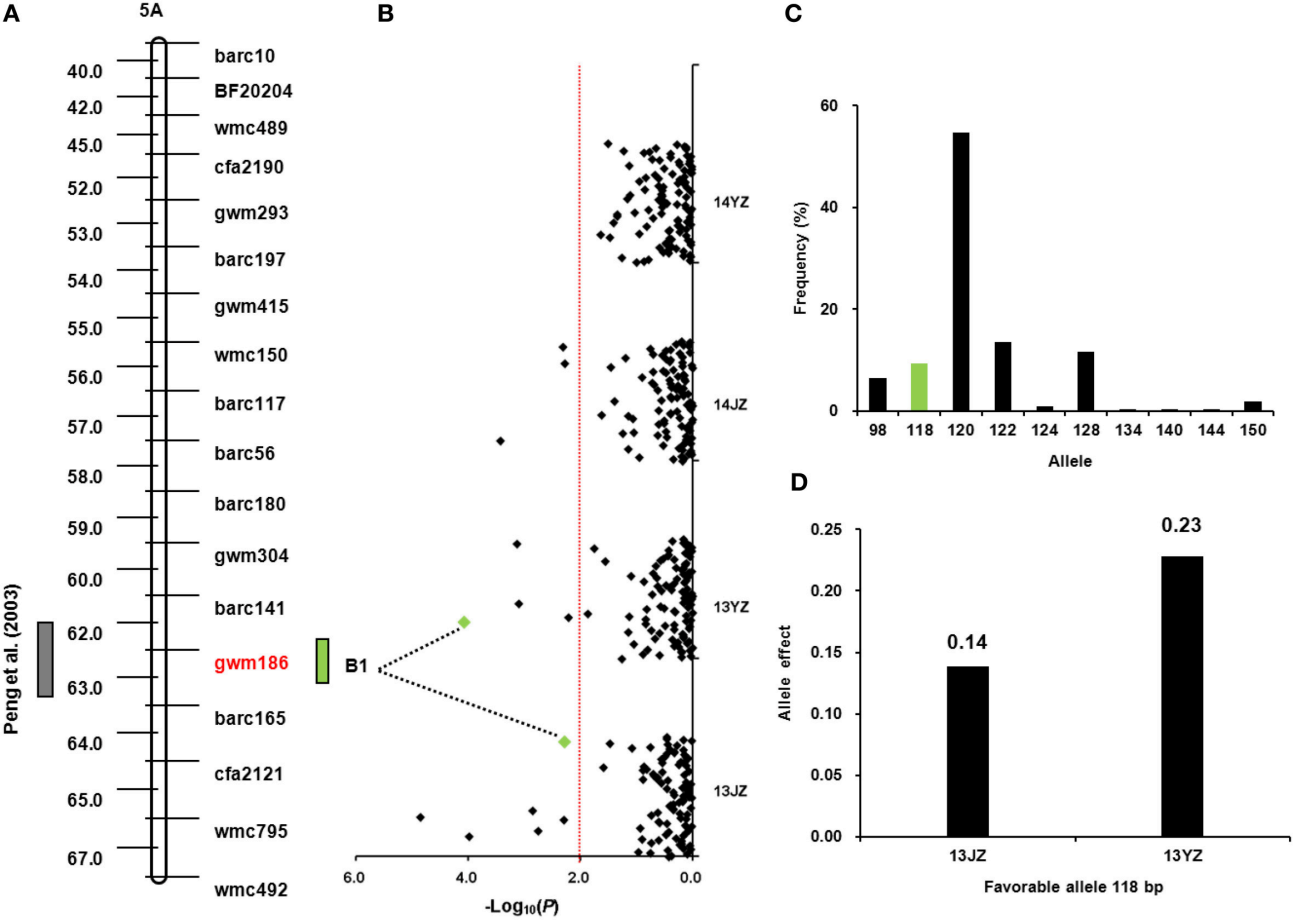
**Favorable allele of *Xgwm186* and genetic effect on grain-setting in the basal spikelets**. **(A)** QTL locus at *Xgwm186-5AL* (Peng et al., 2003); **(B)** Association signals in the natural population using a mixed linear model (*P* < 0.01); green dots represents the associated signal of *Xgwm186* in different environments; **(C)** Frequency distribution of *Xgwm186-5A* alleles in the population; green bar represents favorable allele of 118 bp; **(D)** Genetic effects of *Xgwm186-5A*_**118**_ in environments 13JZ and 13YZ.

A yield QTL was reported previously at *Xgwm186* on chromosome 5AL (Peng et al., 2003; Figure 3A). The same locus was detected in this study for grain-setting rate in GNBS1 in environments 13JZ and 13YZ (Figure 3B). The superior allele had 118 bp and its frequency in the population was 9.35% (Figure 3C). It conferred the highest genetic effect among those affecting the first basal spikelet (Table S4) and increased grain number by 0.14 and 0.23 in environments 13JZ and 13YZ, respectively (Figure 3D).

**Figure 4:**
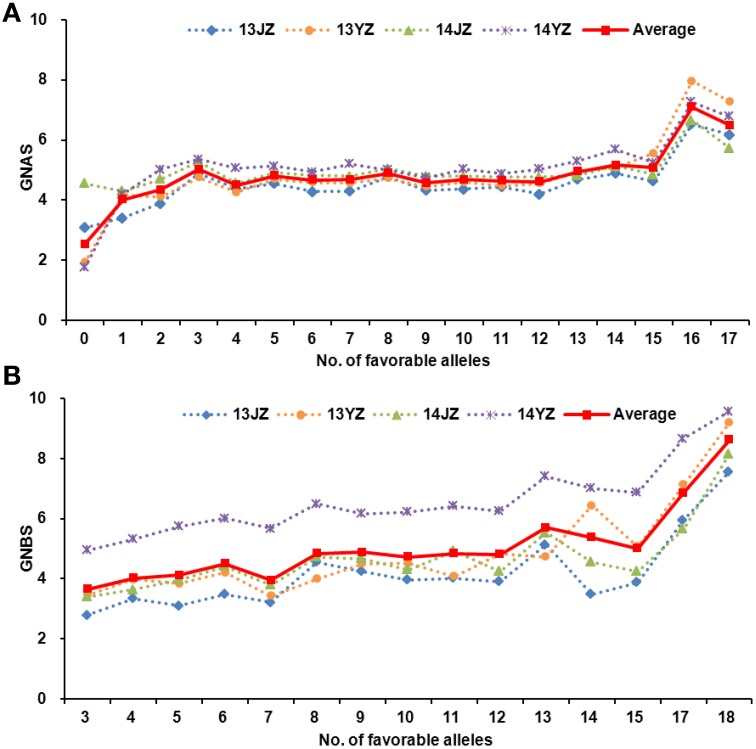
**Relationships between grain numbers in apical and basal spikelets and the number of favorable alleles**. **(A)** The relatedness between the grain numbers in apical spikelets and number of favorable alleles. **(B)** The relatedness between the grain numbers in basal spikelets and number of favorable alleles.

The relationship between favorable allele number and grain-setting rates in both apical and basal spikelets in the entire population is portrayed in Figure 4. In general, higher numbers of favorable alleles led to higher grain-setting rates in apical and basal spikelets; for example, Ningmai 9, Ning 0569, and Yangmai 18 (an offspring of Ningmai 9) carried relatively high numbers of favorable alleles at 34, 33, and 32, respectively (Table S5).

**Figure 5:**
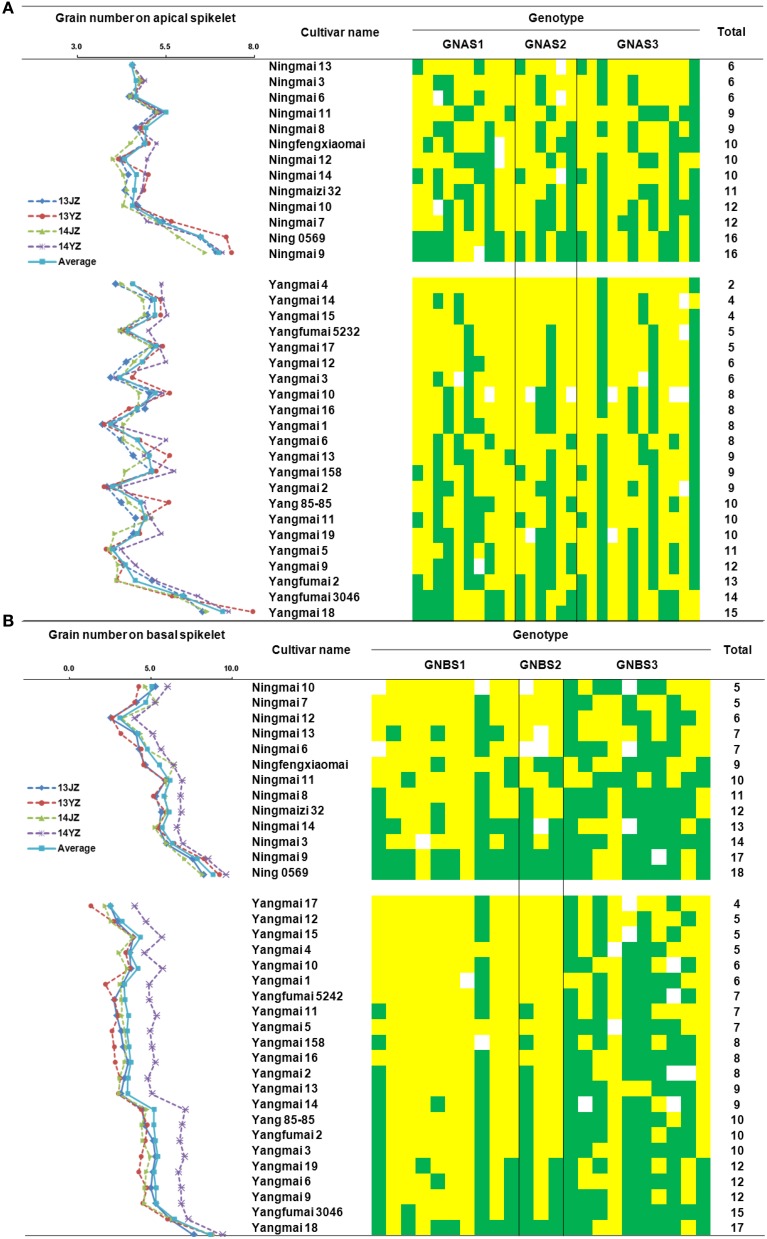
**Distribution of favorable alleles associated with grain numbers in apical and basal spikelets in Yangmai and Ningmai varieties**. **(A)** Distribution of favorable alleles associated with grain-setting in apical spikelets in Yangmai and Ningmai varieties. **(B)** Distribution of favorable alleles associated with grain-setting in basal spikelets in Yangmai and Ningmai varieties.

For rate of grain-setting in apical spikelets, there were 15, 16, and 16 favorable alleles carried by Yangmai 18, Ning 0569, and Ning 9, respectively. Apical spikelet grain-setting rates in those varieties were better than others in the Yangmai and Ningmai series (Figure 5A). Similarly, high grain-setting rates also occurred in basal spikelets of Yangmai 18, Ningmai 9, and Ning 0569 with 17, 17, and 18 favorable alleles, respectively (Figure 5B).

### Dissection of differences in grain-setting rates in apical and basal spikelets between yangmai and ningmai varieties

**Figure 6:**
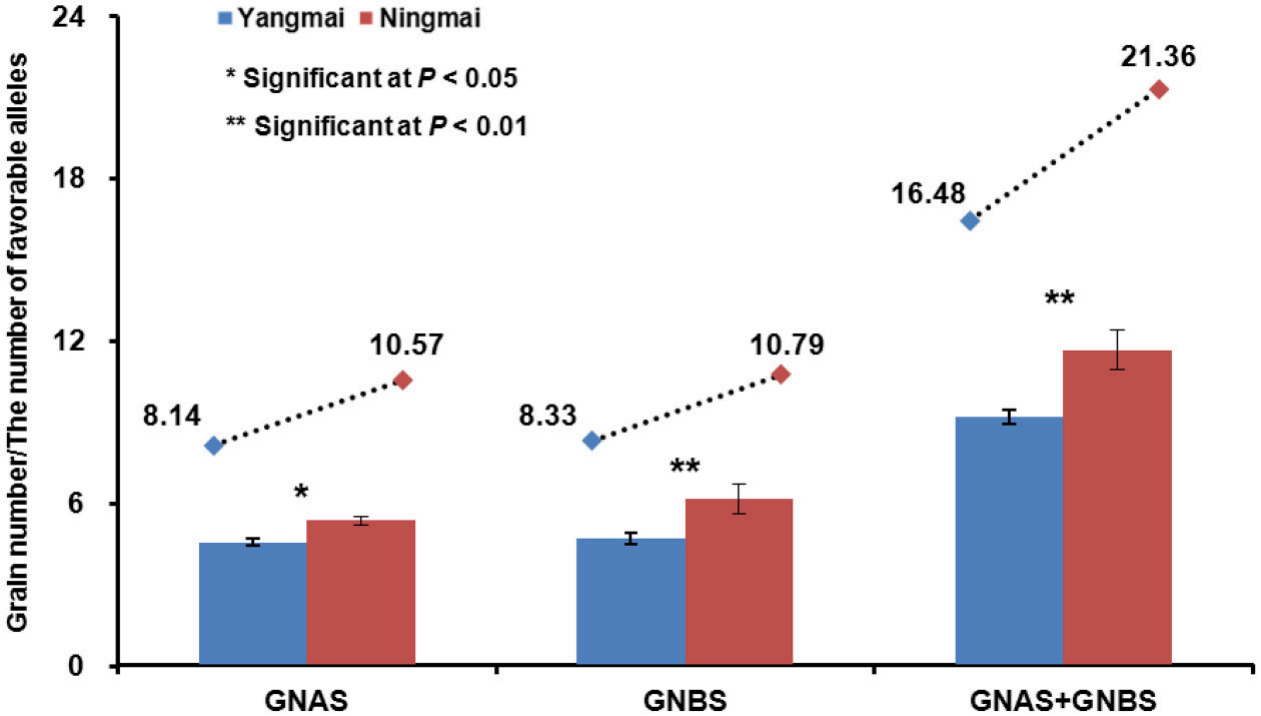
**Relationships between average grain-setting rates in apical and basal spikelets, and numbers of favorable alleles**.

In the Yangtze River region Yangmai varieties predominate over Ningmai varieties although both groups occupy large areas. Grain-setting rates in the Ningmai group were higher than the Yangmai series (Table S5). Apical and basal spikelet grain-setting rates in the four environments were compared between 13 Ningmai and 22 Yangmai varieties. Apart from GNBS in environment 14JZ, the grain-setting rates for the Ningmai series were significantly higher than those for Yangmai series (Figure 6, Figure S4). The numbers of favorable alleles associated with grain-setting in apical and basal spikelets, and the sums of both in the Ningmai series (10.57, 10.79, 21.36) were higher than for the Yangmai series (8.14, 8.33, 16.48), respectively (Figure 6).

## Discussion

### Grain-setting rates in apical and basal spikelets in wheat

Grain yield of wheat will be enhanced if the KNPS is increased by 1 or 2 while other yield factors remain constant (Arisnabarreta and Miralles, 2006; Acreche et al., 2008). There are several ways to increase KNPS; for example, by reducing plant height (Rebetzke and Richards, 2000), increasing spikelet number per spike (Rawson, 1970), and decreasing tiller number (Guo and Schnurbusch, 2015). The approach of the present study was to increase kernel numbers by increasing the numbers of grains in apical and basal spikelets. Significant correlations were shown between grain numbers in the apical and basal spikelets and total numbers per spike (*P* < 0.01) (Figure 1, Table S2). The results showed different grain-setting rates between apical and basal spikelets. Similar numbers of grains were produced in all apical spikelets. However, a significant factor was that the grain-setting rate was especially low for GNBS1 (Table 1). Our results thus confirmed that grain-setting rate in basal spikelets has high potential for increasing the KNPS.

There are relatively few studies on heritability of grain-setting rates. Maich and Zumelzú (1998) reported that narrow-sense heritability of spikelet fertility was 23% using an F_2:3_ population of hexaploid triticale. According to Martino et al. (2015) spikelet fertility is a moderately heritable trait. In the present study broad-sense heritability of grain-setting in apical and basal spikelets was 18.68–21.00% and 9.44–16.37%, respectively. Owing to the low heritability it would be difficult to obtain rapid progress by phenotypic selection at the early stages of a breeding program.

### Analysis of the grain-setting rates in apical and basal spikelets partitions variation in the kernel number per spike

Past QTL analyses of spikelet fertility focused on overall kernel numbers per spike (Slafer and Andrade, 1989; Li et al., 2007; Ma et al., 2007; Wu et al., 2012; Xu et al., 2014). In this study we addressed grain numbers in apical and basal spikelets and analyzed the genetic effects using 106 SSR markers. Thirty two markers were associated with grain-setting rate in apical spikelets and 34 were associated with grain-setting rate in basal spikelets (Table 2, Figure S2). Zhang et al. (2012) reported associations of KNPS with *Xwmc24-1A, Xcfd52-5D*, and *Xgdm127-6D*. In our study these loci were also related to grain numbers in apical spikelets (Table 2). Guo et al. (2015) found associations of *Xgwm337-1D, Xgwm108-3B, Xgwm186-5A, Xgwm415-5A, Xgwm234-5B*, and *Xgwm55-6D* with spikelet fertility, and again these loci were associated with grain numbers in basal spikelets in the present work. Thus, high spikelet fertility might be the consequence of higher grain-setting rate in basal spikelets. Instead, a gene near the *Xgwm102-2D* locus may contribute to grain number in the apical spikelets there by reducing spikelet infertility (Guo et al., 2015). Therefore, some SSR loci reported for KNPS may play a role through regulation of grain-setting rates in apical and basal spikelets. Thus, a significant part of the genetic basis of increased grain-setting per spike may be related to the fertility of apical and basal spikelets.

### Prospects for molecular breeding for kernel number per spike in wheat

The germplasm in this study included several Yangmai and Ningmai varieties. Despite the greater use of Yangmai vaieties by farmers in the Yangtse River region, grain-setting rates in apical and basal spikelets were higher in Ningmai varieties than in Yangmai varieties (Figure 4). Thus, improvement of KNPS in Yangmai varieties should improve yields. Overall, the relatedness of the phenotype and the number of favorable alleles carried by 220 varieties showed that the grain-setting rates in apical and basal spikelets tended to increase with increasing numbers of favorable alleles (Figure 4). Heritability studies of grain-setting rates provided evidence of simple additive effects, consistent with previous results (Salman and Heyne, 1987; Mirabella et al., 2015). Of all varieties Ning 0569 and Ningmai 9 carried the highest numbers of favorable alleles (32 and 34, respectively) associated with grain-setting rates in apical and basal spikelets (Table S5). Therefore, Ning 0569 and Ningmai 9 could be suitable donors of genes for high grain-setting in wheat breeding.

So far, relatively few genetic studies have been conducted on grain-setting rates in wheat, and almost no effective method exists for solving issues of grain-setting rates in the apical and basal spikelets. In this study, 36 SSR loci were associated with grain numbers in apical and basal spikelets (Table 2). To some extent, the results also revealed the basis of higher KNPS in Ningmai varieties compared to the Yangmai group (Figure 6). *Xgwm102-2D, Xgwm108-3B, Xgwm186-5A, Xgwm415-5A, Xcfd52-5D*, and *Xgwm55-6D* were associated with multiple traits in multiple environments. These loci were reported previously to be associated with grain-setting QTLs on a total spike basis (Peng et al., 2003; Zhang et al., 2010a, 2012, 2014; Guo et al., 2015). Among favorable alleles associated with grain-setting rates in apical and basal spikelets the largest effects were conferred by *Xcfa2153-1A*_**202**_, *Xgwm186-5A*_**118**_, *Xgwm156-3B*_**319**_, and *Xgwm537-7B*_**210**_ and their frequencies within the population were 6.51, 9.35, 6.13, and 22.12%, respectively. Low frequencies indicate that particular favorable alleles have not been strongly selected in past wheat breeding. Therefore, to achieve high grain-setting rates more favorable alleles can be introduced to breeding populations and could be tracked by specific molecular markers.

The results from this study were based on a larger mass of phenotypic data obtained through association analysis between grain-setting rates in apical and basal spikelets and SSR markers. In genotypic analysis results may be constrained by limited marker types and marker number. The high density 90K iSelect SNP chip (Wang et al., 2014) could be used to identify further loci associated with grain-setting rates in apical and basal spikelets. Meanwhile, Ning 0569 and Ningmai 9 can be used as donors for accumulating QTLs in breeding lines (Lu et al., 2010; Korir et al., 2013).

## Author contributions

Conceived and designed the experiments: JG, CH, and SC. Performed the experiments: JG, WS, YX. Analyzed the data: JG and CH. Contributed reagents/materials/analysis tools: YZ, BZ, XC, KC, and XZ. Wrote the manuscript: JG, JZ, and CH.

## Funding

This work was supported by grants from the Chinese Ministry of Science and Technology (2011CB100100), the China Agricultural Research System (CARS-3-1-1), the National High-Tech R & D Program of China (2012AA101105), the Six Talent Peaks Project in Jiangsu Province (2013NY-049), and the Key R & D Program in Jiangsu Province (BE2015352-3).

### Conflict of interest statement

The authors declare that the research was conducted in the absence of any commercial or financial relationships that could be construed as a potential conflict of interest.

## Supplementary material

Figure S1 **A schematic representation of the grain set on apical and basal spikelets**.

Figure S2 **Associations of seven phenotypic traits with 106 SSR markers illustrated as dot plots of compressed MLM at *P* < 0.01**. The red dotted line indicates the threshold value of significant association.

Figure S3 **Distribution of pairwise kinship coefficients among 220 bread wheat cultivars based on 106 whole genome SSR markers**.

Figure S4 **The grain number of the apical and basal spikelets among different environments in series of cultivars named Yangmai and Ningmai**.

Table S1 **Information of 220 wheat accessions used in this study**.

Table S2 **Pearson's correlation coefficients between phenotypic traits in the different environments**.

Table S3 **Allele number, MAF, and PIC of 106 polymorphic SSR markers detected in the association panel**.

Table S4 **Favorable alleles and genetic effects of 56 SSR loci significantly (*P* < 0.01) associated with seven phenotypic traits**.

Table S5 **The number of favorable alleles carried by 220 wheat accessions used in this study**.

## Abbreviations

KNPS: Kernel number per spike
GNAS1: Grain number in the first apical spikelet
GNAS2: Grain number in the second apical spikelet
GNAS3: Grain number in the third apical spikelet
GNBS1: Grain number in the first basal spikelet
GNBS2: Grain number in the second basal spikelet
GNBS3: Grain number in the third basal spikelet.
