# Supplementary figures and images for: Association Analysis of Grain-setting Rates in Apical and Basal Spikelets in Bread Wheat (Triticum aestivum L.)
Source: Front Plant Sci. 2015 Nov 20;6:1029. doi: 10.3389/fpls.2015.01029 (PMC4653486; doi:10.3389/fpls.2015.01029)

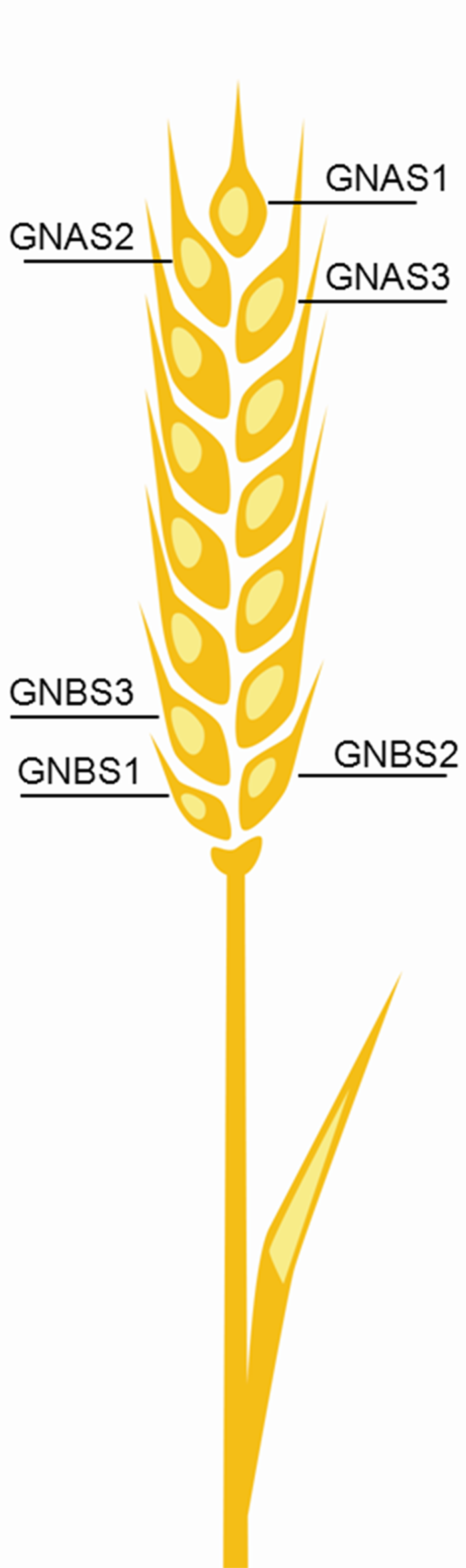

Supplement: Figure S1 — A schematic representation of the grain set on apical and basal spikelets. [file Image1.TIF]

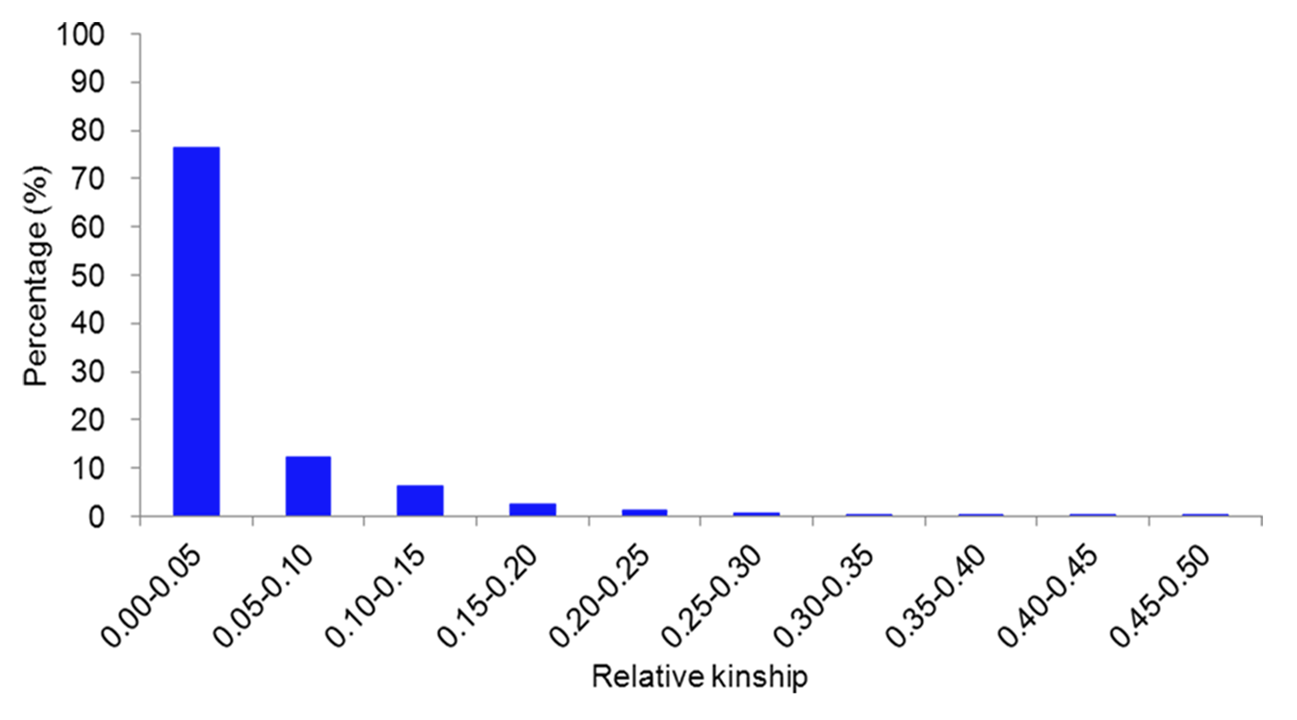

Supplement: Figure S2 — Associations of seven phenotypic traits with 106 SSR markers illustrated as dot plots of compressed MLM at P < 0.01. The red dotted line indicates the threshold value of significant association. [file Image2.TIF]

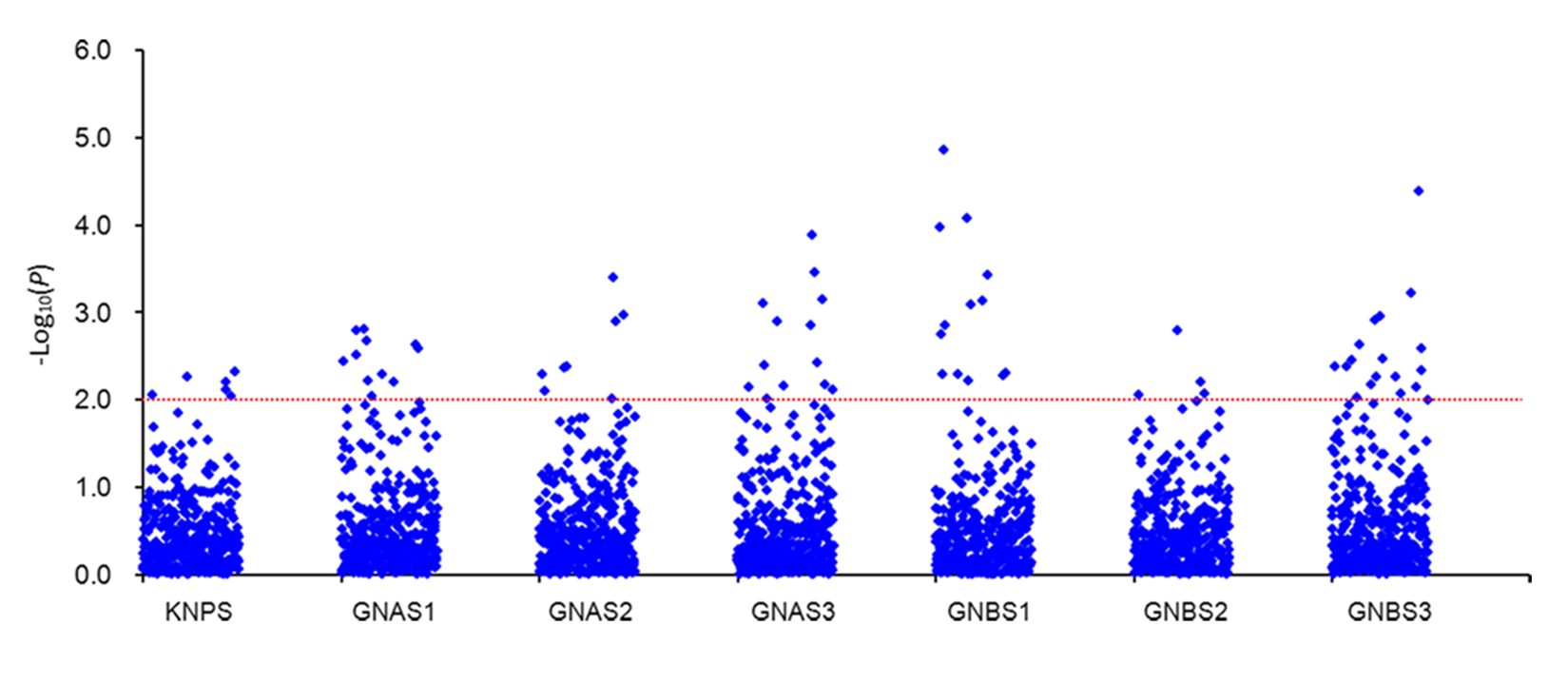

Supplement: Figure S3 — Distribution of pairwise kinship coefficients among 220 bread wheat cultivars based on 106 whole genome SSR markers. [file Image3.TIF]

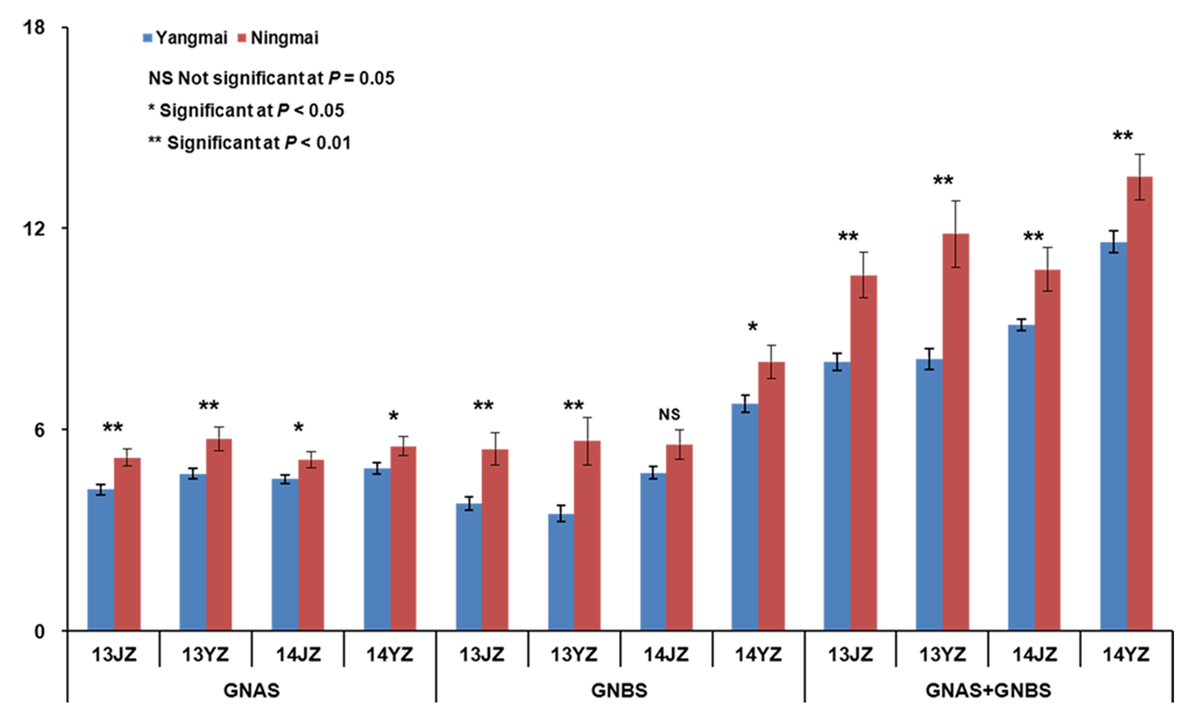

Supplement: Figure S4 — The grain number of the apical and basal spikelets among different environments in series of cultivars named Yangmai and Ningmai. [file Image4.TIF]
